# Inhibitors of the renin-angiotensin system ameliorates clinical and pathological aspects of experimentally induced nephrotoxic serum nephritis

**DOI:** 10.1080/0886022X.2018.1533867

**Published:** 2018-11-07

**Authors:** M. E. Ougaard, H. E. Jensen, I. D. Thuen, E. G. Petersen, P. H. Kvist

**Affiliations:** a Haemophilia PK & ADME, Novo Nordisk, Frederiksberg, Denmark;; b Department of Veterinary Disease Biology, University of Copenhagen, Frederiksberg, Denmark

**Keywords:** Nephrotoxic serum nephritis, pathology, angiotensin-converting enzyme inhibitor, angiotensin II receptor blocker

## Abstract

**Introduction:** Chronic kidney disease (CKD) is a global health concern, but the current treatments only slow down the progression. Thus an improved understanding of the pathogenesis and novel treatments of CKD are needed. The nephrotoxic nephritis (NTN) model has the potential to study the pathogenesis of CKD as it resembles human CKD. The classical treatments with angiotensin II receptor blocker (ARB) or the angiotensin-converting enzyme inhibitor (ACE I) have shown a clinical effect in CKD.

**Methods:** We characterized the disease development in the NTN model over 11 weeks by investigating functional and histopathological changes. We tested doses of 15 and 30 mg/kg/day enalapril and losartan in the NTN model in order to investigate the effect of inhibiting the renin-angiotensin-system (RAS).

**Results:** The NTN model displayed albuminuria peaking on days 6–7, mesangial expansion (ME), renal fibrosis, inflammation and iron accumulation peaking on day 42. However, albuminuria, ME, renal fibrosis and inflammation were still significantly present on day 77, suggesting that the NTN model is useful for studying both the acute and chronic disease phases. Enalapril and losartan significantly enhanced the glomerular filtration rate (GFR) and decreased albuminuria, ME, renal fibrosis and inflammation of NTN-induced kidney disease in mice.

**Conclusions:** This is the first study showing a comprehensive pathological description of the chronic features of the murine NTN model and that inhibiting the RAS pathway show a significant effect on functional and morphological parameters.

## Introduction

Chronic kidney disease (CKD) is characterized by a progressive decline in kidney function, renal inflammation and fibrosis that eventually affects the whole kidney with the final consequence of end-stage renal disease [1]. Various animal models have been established in order to understand the pathogenesis of CKD. The NTN model is a well-known model of acute glomerulonephritis, but it also resembles the chronic stages of CKD [2]. Another advantage is that the model is easy to induce as it is a non-surgical and non-transgenic model. Despite that the NTN model has been utilized for several decades, the knowledge is limited of the disease progression over time regarding clinical and pathological changes, beyond the classical study timeframe of 3–6 weeks [3,4].

A common target of classical treatments of CKD is angiotensin II (Ang II). Intrarenal Ang II regulates the blood pressure by causing vasoconstriction and increasing aldosterone secretion which stimulates the reabsorption of water and sodium [5]. Moreover, in kidney disease, the activation of the renin-angiotensin-system (RAS) and its key player Ang II induces recruitment of renal infiltrating inflammatory cells [6]. The Ang II receptor blockers (ARBs) and the angiotensin-converting enzyme inhibitors (ACE I’s) both ameliorate CKD and their use is recommended as first-line agents in diabetic and non-diabetic renal disease with albuminuria by the National Kidney Foundation [7]. However, treatment with ACE I’s and ARB’s only slow down the progression of CKD [7]. Thus improved understanding of the pathogenesis and improved treatments of CKD are warranted. Furthermore, there is limited knowledge of whether and how RAS plays an important role in the pathogenesis of the NTN model. Therefore, we conducted an 11 weeks’ time-course study to characterize the NTN model and to identify the optimal timeframe showing a clear difference between NTN and control mice. The characterization was focused on clinical and pathological changes including the morphology, mesangial expansion (ME), glomerular basal membrane (GBM) thickening, renal fibrosis, iron and complement accumulation, proliferating of renal cells and infiltration of inflammatory cells. Secondly, we conducted a treatment study with an ACE-i (enalapril) and an ARB (losartan) to investigate whether RAS plays a pathogenic role in the NTN model and to identify a clinically relevant positive control for CKD treatment studies in the NTN model. The doses were based on previously published studies treating CKD mice with enalapril or losartan [8–10]. The treatment effect on the pathogenesis was investigated by evaluating several histopathological endpoints and the highly relevant parameter of kidney function; the glomerular filtration rate (GFR).

## Methods

### *In vivo* studies

Twelve-weeks old CD1 mice (Charles River, Germany) were housed in a facility with a 12 h light/dark cycle. All animal experiments were approved by the Danish Animal Inspectorate and the Novo Nordisk ethical review board. Passive NTN was induced by tail-vein injection of 50 μl of sheep anti-rat NTS (Probetex, San Antonio, USA, PTX-001S lot#199-8) for three consecutive days. Control mice received PBS in a similar manner. The study design followed a randomized block design with 40 mice in each group. At days 7, 42, 63 and 77 ten mice per group were sacrificed, and plasma and kidneys were collected. Prior to termination, mice were induced with isoflurane and the kidneys were perfused with 0.9% NaCl with Heparin (10 U/ml).

The treatment study was conducted similarly, but the timeframe was 42 days. The study included 6 groups consisting of a vehicle group (only NTS, *n* = 8), two enalapril groups (15 or 30 mg/kg/day), two losartan groups (15 or 30 mg/kg/day, *n* = 8 and 8, respectively), and a healthy control group (*n* = 8). During the acclimatization the fluid intake of the mice was measured to calculate the doses of enalapril and losartan as it was administrated via their drinking water. The doses were calculated based on water intake and body weight.

### Urine and plasma analysis

Urine samples were collected by metabolic caging for 18 h on days 6–7, 34–35, and 62–63 and on days 7–8, and 36–37 in the treatment study. The urinary albumin concentration was measured by ELISA (Bethyl Laboratories, cat.no. E90-134) and the 24 h urinary albumin excretion rate (UAER) was calculated. The urinary creatinine was measured by a COBAS 8000 analyser ande the albumin creatinine ratio (ACR) was calculated. Blood samples were collected on days 7, 42, 63, and 77 in the time course study and on days 13 and 42 in the treatment study. Renin plasma concentration was measured by ELISA (R&D systems, Minneapolis Cat.no DY008).

### Glomerular filtration rate

The glomerular filtration rate (GFR) was measured by a preclinical transdermal GFR monitor (Medibeacon GmBH, Mannheim, Germany) as previously described [11]. In short, two times two centimeters fur on the back of the mice were depilated 24 h prior to GFR measurements. FITC-sinistrin (Medibeacon GmBH, Mannheim, Germany) was dissolved in physiological saline, and a stock of 15 mg/ml was prepared and stored at –20 °C away from light. Mice were shortly anesthetized while the GFR monitor was adhered to the depilated area. Subsequently, the mice were injected with 7.5 mg/100g BW of FITC-sinistrin intravenously into the tail vein. The mice were placed for one hour in a single-cage. Afterward the tape was removed and the GFR data were transferred and GFR was calculated.

### Histological analysis

The kidneys were fixed in 10% neutral formalin buffer for 30 h, processed by standard procedures and embedded in paraffin.

Scoring of the morphological changes including ME, GBM thickening, metaplasia, hyaline droplets and protein casts as described in Supplementary materials.

H&E and Perls Prussian Blue staining were performed following the standard procedure. Sheep anti-GBM immunohistochemical (IHC) staining is described in Supplementary materials.

### Immunohistochemical characterization of proliferation, fibrosis, inflammation and complement deposition

All IHC stainings are described in Supplementary materials, Table 1.

**Table 1.** A time course evaluation of morphological changes in the passive NTN model.

### Digital image analysis for quantification of Ki-67, Perl, collagen III, CD45, CD3, F4/80 and C3d

The slides were scanned by a Nanozoomer 2.0 (×20). The image analysis was performed using Visiopharm Integrator System (VIS; Visiopharm, Denmark). An automated tissue detection protocol was performed as previously published [12]. Quantification of collagen III, CD45, CD3, F4/80 and C3d was determined in a region of interest (ROI) restricted to the kidney cortex. Within the ROI, a threshold analysis was performed using an HDAB-DAB filter (∞ - 140) (Ki-67), Haematoxylin (∞ - 140) (Perl) or a red-green filter (30–170) (collagen III, CD45, CD3, F4/80 and C3d) as previously published [12].

### Statistics

Statistical analyses were performed using GraphPad Prism (v7.03; GraphPad, CA), and data were presented as mean ± standard deviation (SD). D’Agistino-Pearson and Shapiro–Wilk normality tests were performed. Normally distributed data were analyzed by one-way ANOVA multiple testing with Turkey’s correction, and non-normally distributed data were analyzed using Kruskal–Wallis multiple testing with Dunn’s correction. A *p* value <.05 was accepted as significant.

## Results

### A time course evaluation of clinical measurements of the passive NTN model

**Figure 1.**
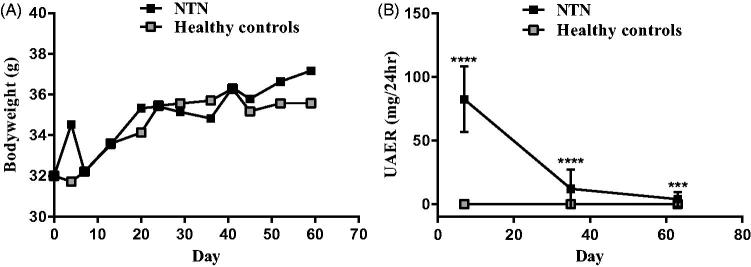
A time course evaluation of clinical measurements of the passive NTN model. (A) XY plot showing body weight (BW) over time. (B) XY plot showing the urinary albumin excretion rate (UAER). Data are shown as mean ± SD. ***p < .001, ****p < .0001, NTN group vs. healthy control group by one-way ANOVA.

At baseline, both the NTN and control mice on average weighed 32 g. After 11 weeks the NTN mice weighed on average 2 g more than the control mice (Figure 1(A)) and did not show compromised health during the 11 weeks.

The NTN mice developed significantly increased urinary albumin excretion rate (UAER) which peaked on days 6–7, but the UAER remained significantly increased until days 62–63 compared to their healthy controls (Figure 1(B)).

### A time course evaluation of morphological changes in the passive NTN model

**Figure 2.**
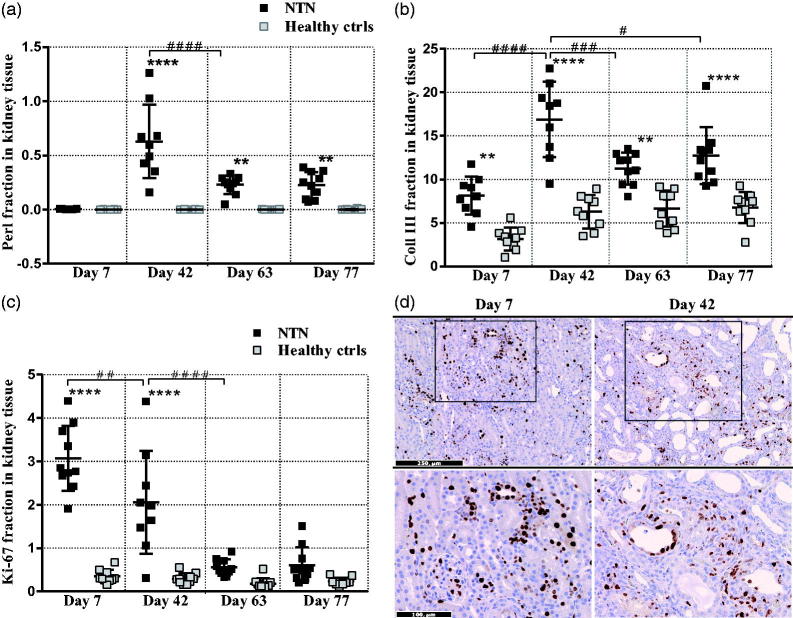
A time course evaluation of morphological changes in the passive NTN model. (A) Scatter plot is showing semi-quantification of Perl positive area in the cortex area. (B) Scatter plot is showing semi-quantification of Collagen III positive area in the cortex area. (C) Scatter plot is showing semi-quantification of the Ki-67 positive area in the cortex area. (D) Representative images are showing the Ki-67 positive area in the cortex area. Data are shown as mean ± SD. **p < .01, ****p < .0001 NTN groups vs. healthy control groups and, #*p* < .05, ##*p* < .01, ###*p* < .001 ####*p* < .0001 NTN group vs. NTN group by one-way ANOVA using Tukey’s multiple comparisons test (*n* = 10).

**Figure 3.**
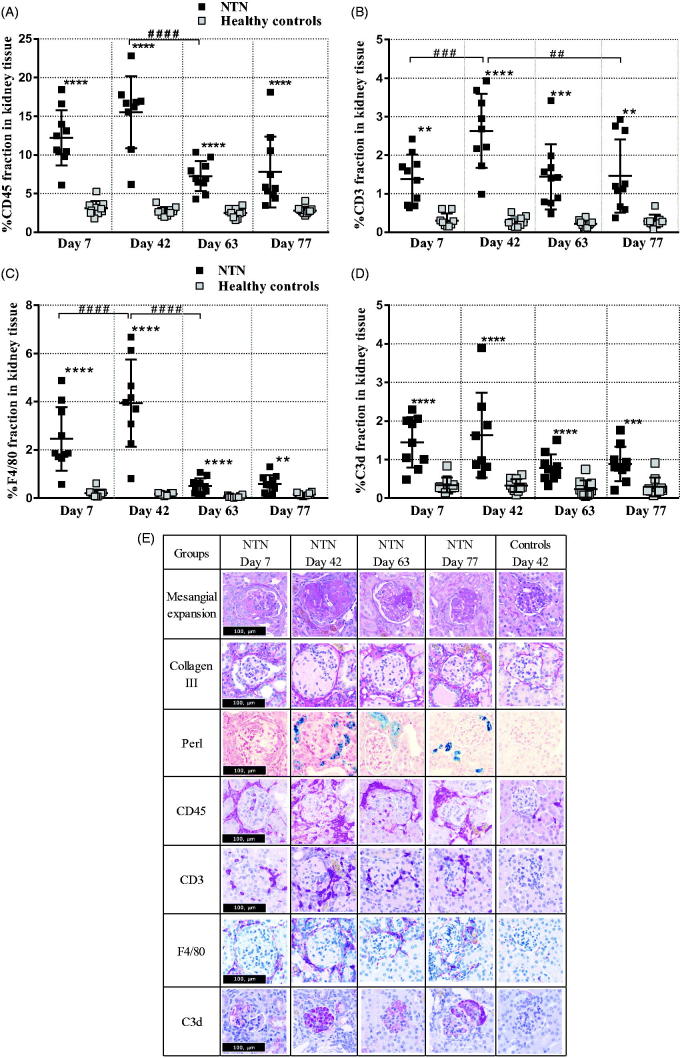
Time course of renal inflammatory response. (A) Scatter plot is showing semi-quantification of the CD45 positive area of the cortex area. (B) Scatter plot is showing semi-quantification of the CD3 positive area of the cortex area. (C) Scatter plot is showing semi-quantification of F4/80 positive area of the cortex area. (D) Scatter plot is showing semi-quantification of the C3d positive area of the cortex area. (E) Representative histopathological images of the disease change over time. Data are shown as mean ± SD. **p < .01, ***p < .001, ****p < .0001 NTN groups vs. healthy control groups and, ##*p* < .01, ###*p* < .001 ####*p* < .0001 NTN group vs. NTN group by one-way ANOVA using Tukey’s multiple comparisons test (*n* = 10).

Antibodies (NTS) (Supplementary materials, Figure 1) were present in all glomeruli and the majority of glomeruli were focally or diffusely enlarged, and intra- and periglomerular infiltration of inflammatory cells was seen at day-7 (Figure 3). At this early stage of the disease, both tubular and glomerular cells proliferate (Figure 2(C–D)), but the glomerular complement deposition (Figure 3(D)) and ME was modest (Table 1 and Figure 3(E)), and tubular changes (Table 1) together with fibrosis were mild or infrequently observed (Figures 2(B) and 3(E)). The hypercellularity of glomeruli had increased significantly at day-42 due to the proliferation of mesangial cells and infiltration of inflammatory cells consisting of neutrophils, macrophages and lymphocytes. There was clear glomerular complement deposition (Figure 3(D,E)) and thickening of the GBM, globally sclerotized glomeruli and some crescent formation in contrast to day-7 (Table 1). Tubular changes were observed focally and characterized by tubular atrophy, i.e., a curvy basal membrane, peritubular infiltration of inflammatory cells, focal tubular casts and PAS-positive intratubular vesicles were observed (Table 1). Iron accumulation had developed at day-42 whereas none was observed at day-7 (Figures 2(A) and 3(E)). The fibrotic changes were significantly increased and located both intra- and extraglomerularly (Figures 2(B) and 3(E)). From day-42, the general histopathological changes seem to decrease but a ME, inflammation and fibrosis were evident in and around glomeruli and tubuli (Table 1, Figures 2 and 3).

### Inhibitors of RAS restores the glomerular filtration rate and ameliorates the renal damage

**Figure 4.**
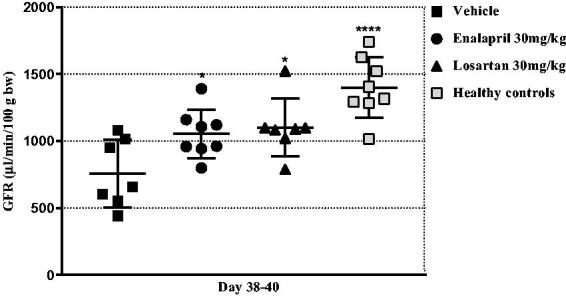
Inhibitors of the renin-angiotensin system improves the kidney function. Scatter plot showing the glomerular filtration rate (GFR) measured on day 38–40. Data are shown as mean ± SD. *p < .05, ****p < .0001 groups vs. vehicle group by one-way ANOVA.

Based on the chronic NTN model in the 77 days study it was concluded that clinical and morphological parameters differed mostly at day-42, therefore, the treatment study with inhibitors of the RAS system was then performed for 42 days. The bodyweight measurements throughout the study showed that neither enalapril nor losartan caused any weight loss in the treated mice (Table 2). The kidney function was evaluated by measuring albuminuria and the GFR. Enalapril and losartan treatment (15 and 30 mg/kg/day) showed significantly reduced albumin concentration and UAER on days 7–8 and 36–37 compared to the vehicle group. Doses of 30 mg/kg/day of losartan showed a significantly improved effect on the UAER compared to 15 mg/kg/day losartan on day 7–8 (19.1 ± 15.0 and 38.0 ± 22.7 (mg/ml), respectively). Both enalapril and losartan (30 mg/kg/day) reduced the ACR to baseline (Table 2). The GFR measurements on days 38–40 showed an effect of treatment with 30 mg/kg/day enalapril and losartan (Figure 4). The effect on the negative feedback action of angiotensin II on renin production was investigated by measuring plasma renin concentrations. Enalapril treatment caused significantly increased plasma renin concentrations which increased over time and losartan-treated mice showed a tendency towards increased plasma renin concentration on days 13 and 42 compared to the vehicle mice (Table 2).

**Table 2:** The effect of inhibitors of the renin-angiotensin system on clinical data including body weight (BW), albumin concentration, urinary albumin excretion rate (UAER), and renin plasma concentration data are shown as mean ± SD.

Groups	Vehicle	Healthy ctrls	Enalapril	Enalapril	Losartan	Losartan
	N = 10	N = 10	15mg/kg N = 10	30mg/kg N = 10	15mg/kg N = 10	30mg/kg N = 10
Bodyweight (g)	Start	Start	Start	Start	Start	Start
	31.2 ± 2.4	31.3 ± 1.8	31.3 ± 2.3	30.8 ± 1.4	30.2 ± 1.4	31.3 ± 0.9
	End	End	End	End	End	End
	33.1 ± 2.7	34.3 ± 2.9	33.5 ± 2.6	32.5 ± 2.2	32.9 ± 1.7	33.7 ± 1.7
Albumin (mg/ml)	Day 7–8	Day 7–8****	Day 7–8***	Day 7–8****	Day 7–8*	Day 7–8****
	30.3 ± 10.4	0.04 ± 0.03	11.8 ± 8.8	8.9 ± 8.8	18.2 ± 9.8	9.9 ± 5.5
	Day 36–37	Day 36–37****	Day 36–37****	Day 36–37****	Day 36–37	Day 36–37****
	6.6 ± 3.6	0.02 ± 0.02	1.1 ± 1.0	1.0 ± 1.4	3.9 ± 3.4	0.8 ± 0.6
Urinary albumin excretion rate (mg/24hrs)	Day 7–8	Day 7–8****	Day 7–8****	Day 7–8****	Day 7–8**	Day 7–8****
	75.0 ± 28.6	0.04 ± 0.02	19.9 ± 18.1	12.6 ± 15.3	38.0 ± 22.7	19.1 ± 15.0
	Day 36–37	Day 36–37****	Day 36–37****	Day 36–37****	Day 36–37***	Day 36–37****
	23.1 ± 16.4	0.04 ± 0.04	2.1 ± 1.8	1.0 ± 11.4	6.4 ± 7.9	1.8 ± 2.9
Albumin creatinine ratio (mg/mmol)	Day 36–37	Day 36–37***		Day 36-37***		Day 36–37***
	4437 ± 2797	320.1 ± 120.3		312.3 ± 469.5		544.6 ± 302.2
Renin (pg/ml)	Day 13	Day 13	Day 13****	Day 13****	Day 13	Day 13*
	23296 ± 6579	15793 ± 3132	59849 ± 14187	62956 ± 20631	42021 ± 11893	43144 ± 9421
	Day 42	Day 42	Day 42****	Day 42****	Day 42	Day 42
	20972 ± 9578	19283 ± 47686	217021 ± 55210	297891 ± 130809	73480 ± 77856	114541 ± 72979

**Figure 5.**
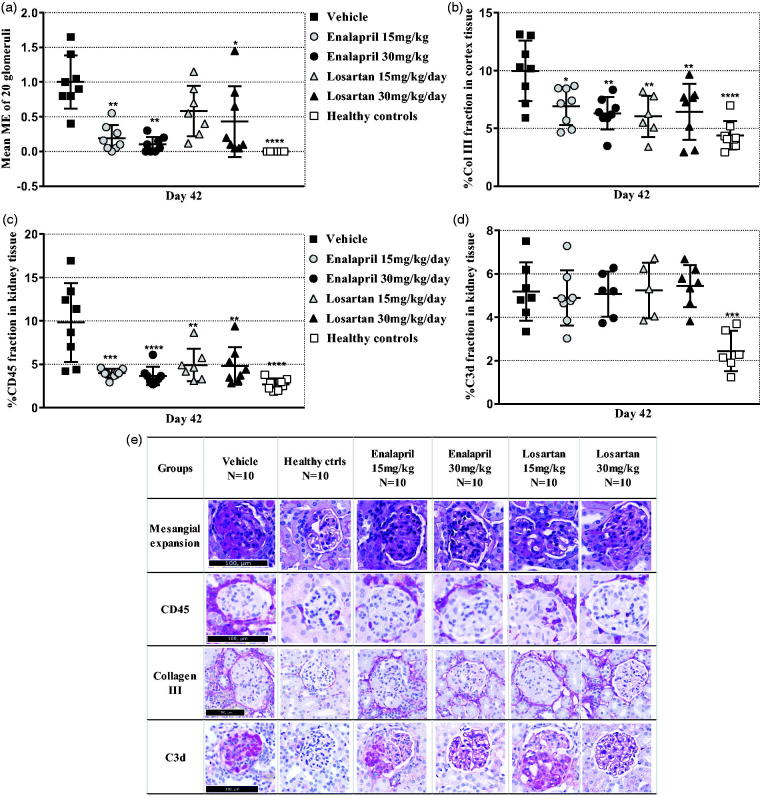
Effect of inhibitors of the renin-angiotensin system on histopathology. (A) Scatter plot is showing the mean glomerular mesangial expansion (ME) score. (B) Scatter plot is showing semi-quantification of collagen III positive area of the cortex area. (C) Scatter plot is showing semi-quantification of the CD45 positive area of the cortex area. (D) Scatter plot is showing semi-quantification of the C3d positive area of the cortex area. (E) Representative histopathological images. Data are shown as mean ± SD. *p < .05, **p < .01, ***p < .001, ****p < .0001 groups vs. vehicle group by one-way ANOVA using Tukey’s multiple comparisons test or Kruskal–Wallis multiple testing (*n* = 10).

The 15 and 30 mg/kg/day enalapril groups showed a significant reduction in the mean ME score compared to the vehicle group. Interestingly, only the 30 mg/kg/day losartan group showed reduced ME compared to the vehicle group (Figure 5(A,E)). Doses of 15 and 30 mg/kg/day of enalapril and losartan reduced the renal accumulation of collagen III on day 42 (Figure 5(B,E)). Furthermore, 15 and 30 mg/kg/day enalapril and losartan reduced the level of infiltration by leukocytes (Figure 5(C,E)). Neither enalapril nor losartan treatment had a significant effect on complement C3d deposition when analyzing glomeruli separately or the whole kidney (Figure 5(D,E)).

## Discussion

The 11 weeks’ time course study was performed to characterize the disease development in detail in acute and chronic settings and to identify the optimal time frame where disease parameters differ the most between diseased and control mice; enabling the optimal point in time for identification of treatment effects in the NTN model. We found that the NTN model exhibits persistence of features of human CKD until day 77 including albuminuria, ME, renal fibrosis, and renal inflammation. The NTS-induced albuminuria has previously been demonstrated to be caused by podocyte loss by nephrin IHC (data not published). However, the observed continuous decline in the albumin concentration and the UAER on days 6–7 indicate that the albuminuria ameliorates over time. It has previously been demonstrated that a glomerulus losing a limited part of its podocytes, the protein leakage from that specific glomerulus is ameliorated [13,14]. Moreover, a major CKD hallmark is declined kidney function evaluated by GFR, which was also observed in the NTN model but it can also be caused by the significant ME that reduces the peripheral capillary surface and the filtration surface [15,16].

The histopathological evaluation showed similarities to subtypes of glomerulonephritis (GN) including ME, GBM thickening, inflammation, tubular casts, renal fibrosis, iron and glomerular complement accumulation [17–19]. Metaplasia describes the substitution with the cuboidal epithelium lining Bowman’s capsule which is believed to be more resistant to local changes in cytokine or extracellular matrix components caused by proteinuria [20,21]. The proliferation of glomerular and tubular cells visualized by Ki-67, is also suggested to be an adaptive response to cellular damage to reduce the loss of filtered proteins [13]. The glomerular protein leakage caused excessive tubular reabsorption resulting in the tubular PAS-positive cytoplasmic vesicles. The excessive reabsorption is hypothesized to lead to tubular degeneration and tubulointerstitial inflammation and fibrosis. Moreover, the tubulointerstitial disease is also believed to be caused by tubular iron accumulation. Due to the glomerular protein leak, iron enters the tubule lumen which previously has been shown to be toxic to the renal tubule [22,23]. Characteristics like glomerular and tubular proliferation, renal fibrosis and iron deposition together with GFR decline were all observed in varying degrees over time in the NTN model suggesting qualitative similarities to CKD in humans.

The improvement of the clinical and pathological findings over time supports that the NTS induced a reversible podocyte loss of the main part of all glomeruli because a more severe podocyte loss causes progressive glomerular injury without resolution. However, not all glomeruli had recovered on day-77 supporting the observation on day-42, that the NTS, besides ME, also induced sclerotic and crescentic glomeruli as these pathological changes unlikely would return to normal functioning glomeruli again [24]. Therefore, the NTS-induced kidney disease seemed to peak on most clinical and histopathological parameters on day 42, where after signs of improvements were observed, but pathological changes consistent with chronic nephritis were still present on day-77.

The clinical effect of inhibiting RAS showed that both enalapril and losartan significantly improved the kidney function by reducing the albuminuria, ACR, and increasing the GFR. The histopathological evaluation showed an effect on ME, as Ang II is believed to induce proliferation of mesangial cells and increase the expression of extracellular matrix (ECM) proteins [25]. Both enalapril and losartan reduced renal accumulation of collagen III supporting the suggestion that locally produced Ang II by proximal tubular cells increases ECM deposition [26]. Furthermore, enalapril and losartan reduced the infiltration of CD45+ cells supporting the suggestion that Ang II promotes immune activation and recruitment of inflammatory cells [26,27]. An important action of enalapril and losartan is that blocking of Ang II affects the local renal hemodynamic regulation, which likely causes an indirect effect on proteinuria, renal inflammation and fibrosis [25,28]. The hemodynamic regulation is possibly caused by activation or upregulation of the protective RAS pathway that includes ACE2 and its product Ang-(1–7) [29]. In experimental models of renal diseases, Ang-(1–7) showed vasodilating, anti-inflammatory and anti-fibrotic effects [30,31]. Both ACE-inhibitors and ARB’s have been demonstrated to increase the circulating levels of Ang-(1–7) in patients with hypertension and CKD. The increased renin excretion, due to the negative feedback mechanism caused by enalapril, causes an increased level of Ang I, which can be converted into Ang-(1–7) by ACE2 and other endopeptidases. ARB’s increase the availability of Ang II that can be converted into Ang-(1–7) by ACE2 [31].

In conclusion, the NTS causes glomerular damage through the binding of antibodies to GBM and thereby induces complement activation, glomerular damage and GBM-thickening with ME. Moreover, significant glomerular proliferation and development of tubular casts, hyaline droplets together with infiltration of inflammatory cells, renal fibrosis and iron accumulation were also observed. The glomerular damage also causes the clinical parameters; albuminuria and GFR decline. Thus, the NTN model displayed acute and chronic functional and morphological characteristics of CKD. Finally, hallmarks of CKD including GFR, albuminuria, ACR, ME and fibrosis were improved by clinically relevant treatments of CKD. Both enalapril and losartan enhanced the kidney function of NTN-induced mice demonstrating the relevance of the NTN model for studying the pathogenesis of acute and chronic phases of kidney disease and for identification of novel CKD treatment strategies.
